# The promise of PD1/PDL1 targeted immunotherapy in locally advanced cervical cancer: a game-changer for patients outcome?

**DOI:** 10.3389/fimmu.2025.1573576

**Published:** 2025-05-13

**Authors:** Fadila Kouhen, Adil El Ghanmi, Hanane Inghaoun, Hayat Miftah, Bouchra Ghazi, Abdallah Badou

**Affiliations:** ^1^ Mohammed VI Faculty of Medicine, Mohammed VI University of Sciences and Health (UM6SS), Rabat, Morocco; ^2^ Laboratory of Neurooncology, Oncogenetic and Personalized Medicine, Faculty of Medicine, Mohammed VI University of Sciences and Health (UM6SS), Casablanca, Morocco; ^3^ Department of Radiotherapy, International University Hospital Cheikh Khalifa, Casablanca, Morocco; ^4^ Immunopathology-Immunotherapy-Immunomonitoring Laboratory, Faculty of Medicine, Mohammed VI University of Sciences and Health (UM6SS), Casablanca, Morocco; ^5^ Department of Gynecology and Obstetrics, Mohammed VI International University Hospital, Bouskoura, Morocco; ^6^ Laboratory of Immunogenetics and Human Pathologies, Faculty of Medicine and Pharmacy, Casablanca, Morocco

**Keywords:** cervical cancer, chemoradiation, immunotherapy, immune checkpoint inhibitors, clinical trial, biomarkers, personalized treatment

## Abstract

Locally advanced cervical cancer remains a significant therapeutic challenge, with high rates of recurrence and metastasis despite advances in chemoradiation. Immunotherapy, particularly immune checkpoint inhibitors targeting the PD-1/PD-L1 axis, has emerged as a promising strategy to enhance treatment efficacy. This review explores the integration of immunotherapy with standard chemoradiation, highlighting the potential of PD-1 inhibitors, such as pembrolizumab, in improving progression-free survival (PFS) among high-risk patients. Furthermore, the role of predictive biomarkers, including microsatellite instability (MSI) and tumor mutational burden (TMB), is examined to refine patient selection and personalize therapeutic approaches. Emerging strategies, including the use of nivolumab, ipilimumab, and maintenance immunotherapy, are also discussed. While preliminary clinical data are encouraging, further research is required to optimize treatment combinations, establish robust patient selection criteria, and enhance long-term outcomes in cervical cancer management.

## Graphical Abstract

GRAPHICAL ABSTRACT

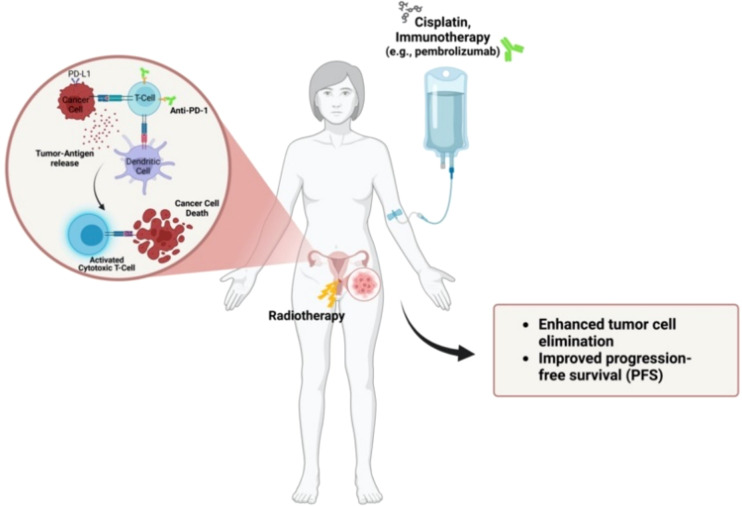

## 1 Introduction

Cervical cancer, particularly in its locally advanced stages, remains one of the most challenging cancers to treat. While significant advances have been made in early detection and treatment modalities, the prognosis for patients with advanced cervical cancer remains poor (1, 2). The primary treatment for cervical cancer involves a combination of chemoradiation, which includes the use of cisplatin-based chemotherapy alongside external beam radiation (3, 4). Chemoradiation has proven effective in reducing tumor burden, improving survival rates, and in many cases, achieving remission. However, despite these advances, a significant proportion of patients still experience recurrence or metastasis. This underscores a crucial limitation of current therapeutic approaches and the need for more targeted and effective interventions to improve long-term outcomes for these patients.

The rationale for integrating immunotherapy in cervical cancer treatment regimens is deeply rooted in the intricate interactions between the host immune dynamics, human papilloma virus (HPV), and cervical cancer cells (5). Because of its viral origin, as most cases are driven by infection with HPV, cervical cancer exhibit a specific immune profile associated in 20% of cases with high tumor mutational burden (6). High-risk HPV types cause 90-100% of cervical cancer, HPV 16/18 causes 70% of cervical cancer, and HPV 16 alone causes 50% of cervical cancer (7). It is noteworthy that even if the majority of cervical cancer are caused by HPV, a considerable percentage of cervical cancer cases (range 8.4–13.9%) are HPV independent (8, 9). Furthermore, HPV independent cervical tumors are associated with advanced stages and worse clinical outcomes (10–15).

Persistent high-risk human papillomavirus (HPV) infection, particularly types 16 and 18, is the main driving force of cervical carcinogenesis. The viral oncoproteins E6 and E7 promote malignant transformation and immune evasion by inactivating the tumor suppressor proteins p53 and retinoblastoma (pRb) (16, 17). The tumor cells infected with high-risk HPV downmodulate the expression of MHC class I, which allows them to escape recognition by cytotoxic T-cells (18). Furthermore, it has been shown that PD-L1’s expression is regulated by E6 protein through a miR-143/HIF-1a axis (19).

The immunosuppressive microenvironment within cervical cancer, characterized by regulatory T cells (Tregs) and myeloid-derived suppressor cells (MDSCs), makes it an excellent target for immunotherapy (18). Clinical trials have demonstrated that immune checkpoint inhibitors (ICIs) can restore T-cell activity and enhance antitumor immunity by targeting PD-1/PD-L1 and CTLA-4 (20–24).

Recently immune signatures for treatment prediction and cervical cancer prognosis have been established (25–29). The potential response predictors identified include, PD-L1-immunoreactive (IR) area, PD-L2, CD8, FGF-basic, IL-7, IL-8, IL-12p40, IL-15, and TNF-alpha (30). It has been shown that impaired TILs and disturbed immune mediators are prominent contributor to therapeutic failure (30). Indeed, patients belonging to the responders group show increased TILs with functional polarization of CD4 T cell populations; Th1, Th2, Th17, and Treg. However, non-responders group exhibit elevated PD-1 scores, CD8+ and PD-L2+ TILs, CD68+ macrophages, and PD-L1 immune reactivity.

In this review we shed light on the potential of immunotherapy-based treatment in locally advanced cervical cancer. Additionally, we cover recent advances in identifying biomarkers for cervical cancer diagnosis and prognosis. We also present a succinct summary of emerging immunotherapy approaches. Overall, this review emphasizes the current standing of immunotherapy in locally advanced cervical cancer.

## 2 Immunotherapy: a promising strategy for cervical cancer treatment

In recent years, the incorporation of immunotherapy into treatment regimens has emerged as a promising strategy in the fight against cervical cancer (31). Immunotherapy aims to enhance the body’s natural immune response to cancer cells, which are often able to evade detection by the immune system. This evasion occurs through various mechanisms, including the upregulation of immune checkpoint pathways that suppress immune activity (32). The immune microenvironment in cervical cancer is shaped by the interplay between HPV-induced inflammation, immune suppression, and the tumor’s ability to evade immune surveillance (33, 34). This environment often includes the accumulation of regulatory T-cells (Tregs), myeloid-derived suppressor cells (MDSCs), and tumor-associated macrophages (TAMs), all of which contribute to the suppression of antitumor immune responses (35). The presence of these immunosuppressive cells within the TME limits the effectiveness of conventional therapies and presents a barrier to successful treatment.

One of the key immune checkpoints involved in this process is the PD-1/PD-L1 axis, which is upregulated in response to persistent HPV infection (36, 37). Tumor cells exploit this pathway to suppress T-cell activation, preventing the immune system from attacking the tumor effectively. By blocking PD-1 or PD-L1, immune checkpoint inhibitors can reverse this suppression, restoring the ability of T-cells to mount an effective anti-tumor response. Clinical studies have demonstrated that PD-1 inhibitors, either as monotherapies or in combination with other treatments, can significantly improve response rates and progression-free survival in patients with metastatic cervical cancer (38).

In addition to PD-1/PD-L1 inhibitors, other immunotherapy strategies, such as immune checkpoint inhibitors targeting CTLA-4, have also been explored in cervical cancer (39). While PD-1/PD-L1 inhibitors have garnered the most attention, the potential for combining different immune checkpoint inhibitors to overcome multiple mechanisms of immune evasion is an area of active research.

## 3 Combination of immunotherapy and chemoradiation: synergistic effects

Recent studies have suggested that combining immunotherapy with chemoradiation may enhance the overall anti-tumor immune response in cervical cancer. Cisplatin, the chemotherapy agent commonly used in chemoradiation regimens for cervical cancer, has been shown to have immunomodulatory effects. Cisplatin can increase the presentation of tumor antigens by promoting dendritic cell recruitment to the tumor site and enhancing the activation of CD8+ cytotoxic T-cells (40, 41). This immune activation occurs alongside the direct cytotoxic effects of cisplatin on tumor cells, which leads to tumor cell death and the release of tumor-associated antigens that can stimulate the immune system.

Similarly, radiation therapy, while primarily designed to kill tumor cells directly, also plays a role in shaping the immune response (42). Radiation has been shown to act as an immunomodulator by inducing a process known as “immunogenic cell death” (43). This process leads to the release of danger-associated molecular patterns (DAMPs) that alert the immune system to the presence of tumor cells. Radiation also promotes the infiltration of T-cells into the tumor and enhances the production of pro-inflammatory cytokines, which further stimulate the immune response (44). These effects make radiation therapy an effective complementary treatment to immunotherapy, especially when combined with immune checkpoint inhibitors. Radiation-SI: Un autre point important que nous devons discuter.

These findings suggest that combining immunotherapy with chemoradiation not only enhances the immune system’s ability to recognize and attack cancer cells but also improves the overall therapeutic efficacy of the treatment regimen.

## 4 Novel immunotherapy approaches in locally advanced cervical cancer setting

In cervical cancer, the rationale for the use of immunotherapy is highly compelling and finds its basis in the complex interactions between the immune system, HPV infection, and tumor cells (5). Indeed, the viral-induced cervical cancer present a specific immunologic profile associated with high tumor mutational burden in approximatively 20% of cases, increasing the neoantigen burden and enhancing the immunogenicity of the tumor (6). In addition, the high expression of ICIs (CTLA4, PD1, PDL1) and the presence of tumor-infiltrating lymphocytes (TILs), unveiled the significant immunogenic potential of cervical cancer settings (45, 46).

**Figure 1:**
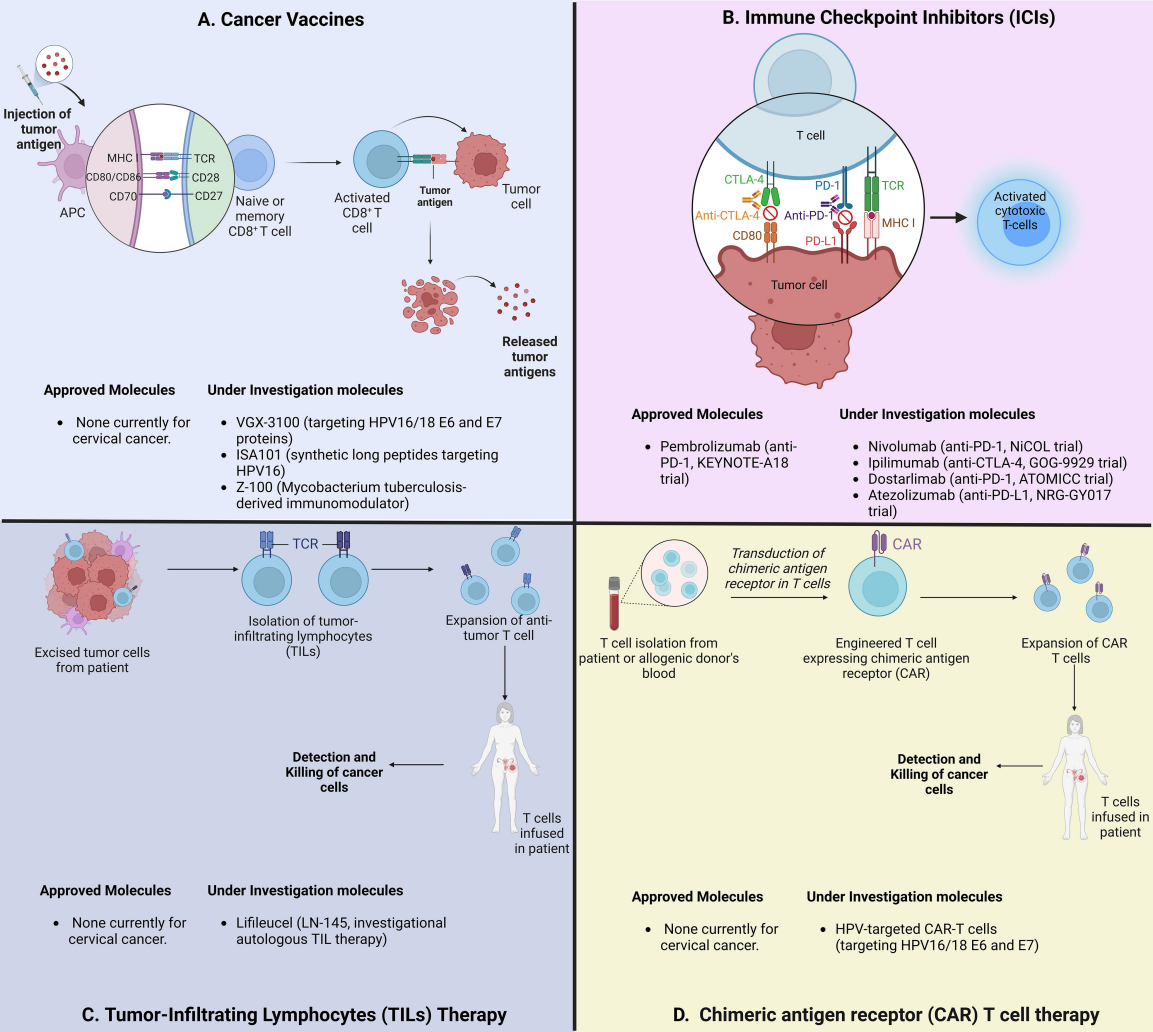
Schematic overview of immunotherapeutic strategies for locally advanced cervical cancer. **(A)** Strategies to induce and enhance cervical cancer-specific T Cells. VGX-3100: HPV16/18 vaccine; Z-100: Mycobacterium tuberculosis extract. **(B)** Strategies to block immune checkpoints and restore T-cell function. PD-1: Programmed cell Death protein 1; CTLA-4: Cytotoxic T Lymphocyte Associated Protein 4. **(C)** Strategies using adoptive cell therapy to augment TILs. **(D)** CAR-T cell therapies targeting HPV-associated proteins.

ICIs has become a central component of first-line treatment for recurrent or metastatic CC, and is under investigation in locally advanced disease with promising results from several phase III trials (47). However, other promising immunotherapeutic strategies are in earlier phases of development, including vaccines (48), tumor infiltrating lymphocytes (49–51), genetically engineered T cells targeting HPV-associated proteins (52–54), and antibody-drug conjugates (55) (**Figure 1**). All these strategies may synergize and change the standard of care for definitive management of the different settings of cervical cancer.

While ICIs represent the most widely studied immunotherapy, therapeutic vaccines and cell-based therapy approaches are under investigation in cervical cancer with encouraging results in pre-clinical trials (48) (**Table 1**). Although these therapies are principally being investigated in metastatic and recurrent diseases, ongoing studies are exploring their benefit in locally advanced cervical cancer (56). The well-known oncogenic properties of HPV and supportive post-infection microenvironment (PIM) created by HPV-infected cells, eagerly promoted investigations on therapeutic vaccines treatment. For cervical cancer, the majority of investigated therapeutic vaccines targeted the oncoproteins HPVs E6 and E7 (57).

**Table 1:** Summary of clinical trials investigating Immunotherapy in Cervical Cancer.

NCT Number	Phase	Immunotherapy Strategy	Patient Population	Current Status	Primary Outcome Measures
NCT05483491	Phase I	**TCR-T Cell Therapy** – KK-LC-1-specific TCR-T cells	Gastric, Breast, Cervical, and Lung Cancer	Recruiting	Maximum tolerated dose (MTD) of KK-LC-1 TCR-T cells (30 days)
NCT04708470	Phase I/II	**Combination Immunotherapy** – Entinostat, PDS01ADC, and Bintrafusp alfa for HPV-associated malignancies	Advanced cancers	Recruiting	ORR of triple combination (2 years), RP2D of entinostat (2 years)
NCT06319963	Phase I/II	**Cancer Vaccine (Lenti-HPV-07)** – Induces immune response against HPV16/18 E6/E7	Cervical, Oropharyngeal Cancer	Recruiting	Safety and Tolerability (12 months), Optimal Biological Dose (28 days)
NCT06640283	Phase II	Biomarker-Based Monitoring – HPV-associated ctDNA detection for cancer surveillance	Cervical and Anal Canal Tumors	Not yet recruiting	Development and validation of a low-cost ctDNA test (2 years)
NCT06358053	Phase I	**TCR-T Cell Therapy** – HPV-16 positive tumor targeting	Advanced HPV-16+ Cancers	Not yet recruiting	Dose-limiting toxicity (28 days), RP2D (2 years), Adverse Events (2 years)
NCT06543576	Phase I/II	**Checkpoint Inhibition + Chemotherapy + Radiation** – PD-1/PD-L1 blockade	Stage IVB Cervical Cancer	Not yet recruiting	Progression-free survival (PFS) (3 years), Incidence of adverse events (30 days)
NCT04574635	Not Applicable	Companion Diagnostic – HPV ctDNA as a predictive biomarker for immunotherapy response	Cervical Cancer	Recruiting	Proportion of patients with undetectable ctDNA posttreatment (6 weeks)
NCT05812677	Phase I	**Oncolytic Virus Therapy** – R130 oncolytic virus targeting tumor cells	Relapsed/Refractory Cervical and Endometrial Cancer	Recruiting	Adverse Events (6 months), Systemic immune response (6 months)
NCT05817214	Phase II	**Dual Immunotherapy** – Cadonilimab + Anlotinib targeting PD-1 and VEGFR pathways	Recurrent/Metastatic/Persistent Cervical Cancer	Recruiting	Objective response rate (up to 2 years)
NCT05914974	Not Applicable	**CRP Biomarker Study** – CRP kinetics as a predictive biomarker for ICI response	Metastatic Gynecological Malignancies	Recruiting	Prognostic value of CRP kinetics under ICI therapy on PFS (10 years)
NCT05805358	Phase II	**Imaging Biomarker Study** – Hyperpolarized 13C MRI for response prediction	Various Cancers	Not yet recruiting	DNP conversion flux (before/after immunotherapy), Clinical PET SUV (before/after immunotherapy)
NCT06737640	Not Applicable	**Biomarker Evaluation** – Association of biochemical/molecular factors with immunotherapy outcomes	Gynecologic Cancer	Not yet recruiting	Evaluation of biomarkers in endometrial and cervical cancer (12 months)
NCT06093438	Phase I/II	**PD-1 Inhibitor + Chemotherapy** – Toripalimab combined with chemotherapy	Locally Advanced Cervical Cancer	Recruiting	Overall response rate (1 year), Volume changes in radiation targets (5 years)
NCT05872724	Phase II	**MRD-Based Adjuvant Therapy** – Monitoring minimal residual disease (MRD)	Postoperative Cervical Cancer	Recruiting	3-year DFS in ITT population
NCT06591078	Not Applicable	**Acupuncture + Immunotherapy** – Enhancing immune response via acupuncture	Cervical Cancer	Not yet recruiting	Objective remission rate (evaluated every 8 weeks, then every 12 weeks)
NCT05475171	Phase II	**Checkpoint Inhibition Induction Therapy** – TRACTION trial	Advanced Cervical Cancer	Recruiting	Overall survival (1 year)
NCT05173272	Phase III	**Induction Chemo + Immunotherapy** – Pre-CRT combination strategy	Advanced Cervical Cancer	Recruiting	Progression-free survival (PFS) (36 months)
NCT06511726	Phase II	**Induction Chemo + Checkpoint Inhibitor** – Cadonilimab plus chemotherapy	Locally Advanced Cervical Cancer	Recruiting	ORR (2 weeks post-induction, up to 18 months)
NCT06641635	Phase III	**Radiotherapy Optimization** – Moderated hypofractionated radiotherapy	Cervical Cancer	Recruiting	Progression-free survival (3 years)
NCT06878222	Phase II	**Dual Checkpoint Inhibitors** – Iparomlimab (PD-1 inhibitor) + Tuvonralimab (CTLA-4 inhibitor)	Neoadjuvant Therapy for Cervical Cancer	Not yet recruiting	Objective response rate (9 weeks), Pathological Complete Response (3 months)
NCT06727617	Phase II	**PD-1 Inhibitor + Chemoradiotherapy** – Serplulimab (PD-1 inhibitor)	Postoperative Cervical Cancer with Risk Factors	Recruiting	DFS (1 year)
NCT06715241	Phase II	**Dual ICI Therapy** – Relatlimab (LAG-3 inhibitor) + Nivolumab (PD-1 inhibitor)	Locally Advanced Cervical Cancer	Recruiting	Objective response rate at 6 weeks (RECIST v1.1)
NCT06416696	Phase II	**PD-1 Inhibitor** – Toripalimab (PD-1 blockade)	High-risk Locally Advanced Cervical Cancer	Recruiting	Progression-free survival (2 years)
NCT05492123	Phase II	**Dual ICI + Chemoradiation** – Nivolumab (PD-1 inhibitor) + Ipilimumab (CTLA-4 inhibitor)	Cervical Cancer	Recruiting	3-year progression-free survival
NCT06238635	Phase II	**Dual Checkpoint Inhibitors** – Dostarlimab (PD-1 inhibitor) + Cobolimab (TIM-3 inhibitor)	Advanced Cervical Cancer	Recruiting	ORR based on irRECIST (up to 2 years)
NCT06095674	Phase I	**Adjunctive Immunotherapy** – ITHACA study (immune modulation strategy)	Cervical Cancer	Not yet recruiting	Dose-limiting toxicity (2 years)
NCT06315257	Phase I	**Cancer Vaccine (PVX7)** – Immunotherapy regimen targeting tumor antigens	Advanced Cervical Cancer	Not yet recruiting	Safety (AEs) (12 months), Feasibility of PVX7 (12 months)

ICI, Immune Checkpoint Inhibitor; ctDNA, Circulating Tumor DNA; MRD, Minimal Residual Disease; Chemo-RT, Chemoradiotherapy; CRT, Concurrent Radiotherapy & Chemotherapy; MTD, Maximum Tolerated Dose; RP2D, Recommended Phase 2 Dose**;** DFS, Disease-Free Survival; OS, Overall Survival; PFS, Progression-Free Survival; ORR, Objective Response Rate; pCR, Pathological Complete Response; CRP, C-Reactive Protein Kinetics; RECIST, Response Evaluation Criteria in Solid Tumors; irRECIST, Immune-Related RECIST; SUV, Standardized Uptake Value; DNP, Dynamic Nuclear Polarization; AE, Adverse Event; SAE, Serious Adverse Event. AE, Adverse Event. Anlotinib, a tyrosine kinase inhibitor that targets Vascular Endothelial Growth Factor receptor (VEGFR). Bintrafusp alfa, bifunctional fusion protein composed of the extracellular domain of transforming growth factor beta receptor II (a TGF-β "trap") fused to a human immunoglobulin G1 monoclonal antibody blocking PD-L1. Cadonilimab, a bispecific antibody targeting PD-1 and CTLA-4. Chemo-RT, Chemoradiotherapy. Cobolimab, humanized IgG4 T cell immunoglobulin and mucin domain-containing protein 3 (TIM-3)-targeted monoclonal antibody. CRP, C-reactive Protein. CRT, Concurrent Radiotherapy & Chemotherapy. ctDNA, Circulating Tumor DNA. DFS, Disease-Free Survival. DNP, Dynamic Nuclear Polarization. Dostarlimab, humanized monoclonal PD-1 blocking antibody. Entinostat, selective class I HDAC inhibitor with potential antineoplastic activity. Hyperpolarized 13C MRI, Hyperpolarized (HP) carbon 13 (13C) magnetic resonance imaging. for response prediction. ICI, Immune checkpoint inhibitors. Iparomlimab, immunoglobulin G4 (IgG4) monoclonal antibody directed against the human PD-1. Ipilimumab, monoclonal IgG1 antibody directed against CTLA-4. irRECIST, Immune-Related Response Evaluation Criteria in Solid Tumors. ITHACA study, ImmunoTHerapy Adjacent to Cervical CAncer (ITHACA) phase I study evaluating the safety and toxicity of a peritumorally injected Balstilimab (PD-1 checkpoint inhibitor), in combination with Botensilimab (a multifunctional Fc-enhanced anti-CTLA-4 CTLA-4 inhibitor), in early-stage cervical cancer. KK-LC-1, Kita-Kyushu Lung Cancer Antigen-1. Lenti-HPV-07, onco-therapeutic lentiviral vector-based vaccines for the treatment of human papillomavirus (HPV)-induced cancers. MRD, Minimal Residual Disease. MTD, Maximum Tolerated Dose. Nivolumab, a humanized anti-PD-1 monoclonal antibody. ORR, Objective Response Rate. OS, Overall Survival. pCR, Pathological Complete Response. PDS01ADC, IL-12 fused antibody drug conjugate. PFS, Progression-Free Survival. Pre-CRT, Preoperative chemoradiotherapy (CRT). PVX7 Cancer Vaccine, a therapeutic prime/boost cancer vaccine consisting of PVX7, which is a combination of two vaccines: (1) the pBI-11 prime vaccine, a therapeutic codon-optimized plasmid DNA vaccine encoding for the E6 and E7 oncoproteins of the human papillomavirus (HPV) subtypes 16 (HPV16) and 18 (HPV18) fused with heat shock protein 70 (HSP70), and (2) the HPV tumor antigen (TA-HPV) boost vaccine, a recombinant vaccinia viral vector-based vaccine encoding epitopes of the E6 and E7 oncoproteins from HPV16 and HPV18. R130, oncolytic HSV-1 R130, a recombinant oncolytic herpes simplex virus type 1 (HSV-1), with potential oncolytic and antineoplastic activities. RECIST, Response Evaluation Criteria in Solid Tumors. Relatlimab, human IgG4 monoclonal antibody directed against Lymphocyte-Activation Gene-3 (LAG-3). RP2D, Recommended Phase 2 Dose. SAE, Serious Adverse Event. Serplulimab, humanized monoclonal anti-PD-1 antibody. SUV, Standardized Uptake Value. TCR, T Cell receptor. Toripalimab, PD-1 blocking monoclonal antibody. TRACTION trial, phase II trial examining the strategy of induction immunotherapy with Lorigerlimab (bispecific antibody targeting CTLA-4 and PD-1) in chemo-naïve cervical cancer patients not amenable to curative treatment. Tuvonralimab, IgG1 monoclonal antibody directed against CTLA-4.

Building on the positive outcome of two phase II studies in metastatic and recurrent settings, the phase III AIM2CERV clinical trial (NCT02853604) was tailored to determine the effectiveness of Axalimogene filolisbac after the completion of CCRT in high-risk locally advanced cervical cancer (FIGO stage I–II with positive pelvic nodes, stage III–IVA, and any stage with para-aortic nodes) (58, 59). Axalimogene filolisbac (ADXS-HPV, Princeton, NJ, USA) is a live attenuated, recombinant Listeria monocytogenes (Lm) bacterium bioengineered to secrete an antigen-adjuvant fusion protein that includes a truncated fragment of listeriolysin O (tLLO) fused to the full-length E7 peptide of HPV-16 (tLLO-HPV-16 E7) (58). The rapid uptake of ADXS-HPV by antigen presenting cells and secretion of the fusion HPV E7 protein, stimulate innate immunity, followed promptly by HPV-specific effector T-cells infiltration of the TME and tumor cell killing (60, 61). Although designed to target HPV type 16-associated cancers, results from ADXS-HPV preclinical and clinical studies showed immunogenic response against high-risk HPV other than type 16 (62). The AIM2CERV clinical trial has been terminated in 2019 and no results have been published yet.

PDS0101 is a multipeptide therapeutic vaccine targeting the E6 and E7 oncoproteins of high-risk HPV type 16. The coadministration of peptides with the nanoparticle R-DOTAP (Versamune) platform, induces type 1 interferons and enhances antigen cross-presentation. PDS0101 induced HPV-specific CD4+ and CD8+ T-cell immune responses and was well tolerated (63). The ongoing single-arm, phase II IMMUNOCERV trial (NCT04580771), focuses on evaluating a liposomal HPV-16 E6/E7 T-cell activating immunotherapy (PDS0101) approach combined with CCRT in advanced cervical cancer patients (64). The interim analysis demonstrated promising results on eight patients that completed the treatment, showing a complete response rate of 87.5% on PET at 3 months; the 1-year disease-free survival rate was 85.7%, and the 1-year overall survival 100%, associated with an acceptable toxicity profile.

Moreover, genomic profiling and radiomic studies to guide the response to immunotherapy in advanced cervical cancer are promising. Accumulated data confirm the dysregulation of molecular pathways like PI3K/AKT/mTOR, PIK3CA, STK11, and PTEN. In patients treated with anti-PD1 immunotherapy, ERBB3 mutation and a high TMB have been shown associated with prolonged survival, making them predictive biomarkers candidates in advanced cervical cancer (65, 66). Radiomic studies hold great potential to guide the response to different immunotherapy regimens, according to intrinsic tumor radiosensitivity (67, 68).

## 5 Challenges of predictive biomarkers for cervical carcinoma immunotherapy

Studies discussed above highlight that only a minority of patients with cervical cancer can benefit from the use of ICI therapy. Improving ICI efficacy can be achieved by identifying potential biomarkers for the assessment of the therapeutic effect. Several factors have been identified as predictive biomarkers for anti-PD1/PDL1 immunotherapy response, including PD-L1 expression in tumor cells and tumor-infiltrating lymphocytes, tumor mutational burden (TMB), microsatellite instability (MSI), and/or mismatch repair deficiency (MMR) (69–71).

Normal cervical tissue does not express PDL1 protein. However, in premalignant and malignant lesions the expression of PDL1 is reported to be in the range of 95% in cervical intraepithelial neoplasia (CIN) and 80% in cervical SCC (72). In cervical SCC, another study reported a low PDL1 expression rate of 24.9% (73). Cervical adenocarcinoma shows low rates of PDL1 expression with one study reporting PDL1 positivity in 14% of samples versus 54% in SCC (74). These discrepancies between studies may be attributed to variations in analysis and detection methods as well as the cut-off values used to define positive expression.

Based on the KEYNOTE-158 clinical trial findings, showing a clinical activity of pembrolizumab in 14% of PDL1-positive patients, the FDA approved pembrolizumab for cervical carcinomas with PDL1 positivity defined by a combined positive score (CPS) ≥ 1 assessed by the DAKO 22C3 assay (75, 76). The CPS calculates the ratio between all PD-L1-positive neoplastic cells, lymphocytes and macrophages, multiplied by 100 and divided by the total number of viable tumor cells (77). However, even if the use of CPS positivity help identifying patients who may respond to PD1/PDL1 blockade, many studies have reported responses in PDL1 negative tumors (78). The heterogeneity in PDL1 expression may contribute to variable results and undermine its reliability as a biomarker (74). More recent approaches for PD1/PDL1 assessment beyond IHC, include PDL1 copy number analysis and RNAish to detect PDL1 mRNA (79, 80).

Tumor mutational burden (TMB) is a surrogate biomarker of neo-antigens load and immunogenicity, with high TMB status associated with favorable response to ICIs (81). The TMB is calculated by assessing the number of nonsynonymous somatic mutations per mega-base (mb) (82–84). High TMB has been shown to be more significantly associated with response to PD-1 and PD-L1 immunotherapy than PD-1 or PD-L1 expression (85). While high TMB is relatively uncommon in cervical cancer, some studies have suggested that patients with high TMB may derive greater benefit from immune checkpoint inhibitors (86). In a study describing the distribution of TMB across a cohort of 284 cervical SCC cases, a TMB median of 5.4 mutations/mb have been calculated, with TMB > 20 mutations/mb in 6.7% cases (85). The potential for combining TMB with other biomarkers, such as PD-L1 expression, to guide treatment decisions is an exciting avenue for future research.

Biomarker analysis of the basket study KEYNOTE 158, based on the companion diagnostic FoundationOne CDx assay, accelerated approval of pembrolizumab in patients with high TMB (≥ 10 mutations/mb) (6). the FoundationOne CDx panel detects substitutions, insertions and deletions, and copy number alterations in 324 genes, select gene rearrangements, and genomic signatures including microsatellite instability and tumor mutation burden (TMB) in patients with advanced or recurrent solid tumors (87). TMB holds significant promise as a predictive biomarker of therapeutic response immunotherapy. Further studies are needed to standardize and harmonize TMB assessment across assays, and confirm its predictive efficacy in diverse settings.

Microsatellite instability (MSI) or mismatch repair deficiency (MMR-d) have been linked to exceptional benefit from ICIs. MSI-high tumors are known to exhibit a higher mutational burden, leading to the production of more neoantigens, which can enhance the immune response to the tumor (88). Although MSI-high tumors are relatively rare in cervical cancer, they have been associated with better responses to immunotherapy in other cancer types, such as colorectal cancer (89, 90). MSI and MMR-d are agnostic indication for the use of pembrolizumab in refractory tumors. In cervical SCC, MSI-high was found in 11.8% (89, 91). Many MSI markers are routinely used to identify MSI-High cancers, with the principal aim of treating patients and offering tailored therapeutic approach. However, the MSI testing is limited to certain types of cancer such as endometrial and colorectal cancer. There is an urgent need to invest in gynecological cancers-specific MSI panels which for more accurate and effective screening (92). A comprehensive characterization of MMR protein expression in cervical cancer confirms that MMR-D is rare compared to neuroendocrine tumors. In a recent study, Van Den Berg et al., propose MSH-2-low and not MMR-D as biomarker of ICI response in cervical cancer due to its association with high mutational burden, immune activation, and RAD50 frameshift mutations (93). In cervical cancer, the identification of MSI-high tumors may offer an opportunity for personalized treatment strategies that incorporate immune checkpoint inhibitors.

A deep understanding of the complex relationship between PD1/PDL1 expression, TMB and MSI status, is a prerequisite to improve the use of immunotherapy in cervical cancer patients.

Tumor-draining lymph nodes (TDLNs) located along the lymphatic drainage pathway of primary tumors, serve as a repository of anti-tumor immune cells and are the primary sites at which anti-tumor lymphocytes are primed to tumor-specific antigens (94). Thus, TDLNs can mirror the immune dynamics of battle between immune and tumor cells. In cervical cancer, immune profiling by flow cytometry of pelvic TDLN revealed predominant and elevated PD-1 expression on effector T-cell subsets. In addition, elevated levels of CD8+ FoxP3+ CD25+ effector T cells were identified as a potential biomarker for predicting response to PD-1 blockade that merits prompt evaluation (95). Gaining knowledge of TDLN-immune biomarkers provides attractive means for improving the evaluation system and developing new avenue of patient stratification, timing of immunotherapy, and efficacy outcomes. Clinical studies should focus on investigating changes in TDLNs during ICIs treatment, including alterations in size, immune cell profiles, and cell function. Additionally, there is a need to enhance diagnostic tools for assessing TDLNs (96).

Given the high financial cost of using predictive biomarker tests in routine practices, prognostic scores based on routine laboratory data represent a promising breakthrough. For example, the Lung Immune Prognostic Index (LIPI) score based on neutrophil, lymphocyte, and LDH levels have been identified as a reliable tool to guide immunotherapy treatment decisions (97).

The identification and validation of cervical cancer biomarkers can be of great importance in designing adaptive clinical trials (98). Unlike non-adaptive trial designs, adaptive designs are flexible using accumulating data based on interim analysis to modify the ongoing trial without affecting the integrity and validity of the trial (99). Recently, several biomarkers were used to guide adaptive trial designs, including the marker-stratified design, marker-strategy design, enrichment design, basket design, N-of-1 design and master protocol design (99).

Finally, effective prevention of cervical cancer lies on early screening. While traditional cervical cytology remains the gold standard for cervical cancer screening, this method is time-consuming, subjective, has limited and highly variable sensitivity, and relies on the expertise and experience of pathologists (100). The emergence of Artificial intelligence (AI) screening systems holds promise in enhancing cervical cancer imaging diagnostics and predicting clinical responses (101). Compared to cytology, AI screening enhances the speed, accuracy, and reliability of cancer detection. AI-enhanced screening relies on consistency of cytopathological results, improving sensitivity, and reducing the risk of misdiagnosis (102, 103). In a study reported by Zhu et al., an AI-aided comprehensive cervical diagnostic system (AIATBS) has been developed (104). This system integrated five AI models; YOLOv3 for object detection, Xception and DenseNet-50 for target classification, U-net for nucleus segmentation, and XGBoost model for final slide-level diagnostic decisions. Although AIATBS system applicability and robustness for routine assistive diagnostic screening was demonstrated, it actually increases the complexity of related-screening processes (101). Rahaman et al., developed a classification of cervical cytology using a deep learning-based hybrid deep feature fusion (HDFF) technique (105). However, the classification focuses only on squamous epithelial cells. Recently, Wang et al., have created an AI-assisted cervical cancer screening (AICCS) by investigating the whole-slide images (WSIs) of cervical cytology (101). Even if the use of AI can enhance the quality of medical services, several limitations need to be considered, mainly limited operator experience, skill levels of cytology preparers, and ethical considerations (101).

## 6 Discussion

The combination of immune checkpoint inhibitors with chemoradiation (CRT) has emerged as a promising therapeutic approach, fueled by the successes observed in malignancies such as non-small cell lung cancer (NSCLC) and small cell lung cancer (SCLC). Notably, durvalumab, an anti-PD-L1 inhibitor, demonstrated significant improvements in both overall survival (OS) and progression-free survival (PFS) following CRT in the PACIFIC and ADRIATIC trials (106, 107), which established its therapeutic potential in lung cancer. This success paved the way for its investigation in cervical cancer, with the CALLA trial marking the first Phase 3 study to evaluate the combination of immunotherapy and CRT for untreated locally advanced cervical cancer (108) (**Table 2**). Published in *The Lancet*, the CALLA trial enrolled a diverse cohort of patients from 105 hospitals across 15 countries, including those with squamous, adenocarcinoma, and adenosquamous histologies. The inclusion criteria encompassed patients with FIGO stages IB2–IIB (with lymph node involvement) or stage ≥III. Participants were randomized to receive either durvalumab (1500 mg every 4 weeks) or placebo alongside standard CRT, which consisted of external beam radiation (45 Gy) with weekly cisplatin or carboplatin, followed by image-guided brachytherapy. The **Table 2** summarizes key clinical trials using Immunotherapeutic strategies in Cervical cancer.

**Table 2:** Summary of completed and ongoing clinical trials using Immunotherapeutic strategies in Locally advanced Cervical Cancer

Trial Name	Phase	Drug	Population	Key Findings/Objectives	Status
**CALLA**	Phase III	Durvalumab	Locally advanced cervical cancer (FIGO stages IB2–IIB with LN involvement, stage ≥III)	No significant improvement in PFS overall; PD-L1 expression may determine response.	Completed
**KEYNOTE-A18**	Phase III	Pembrolizumab	High-risk, locally advanced cervical cancer	24-month PFS: 68% (pembrolizumab) vs. 57% (placebo); OS difference not statistically significant; slightly higher grade 3+ AEs in pembrolizumab group.	Completed
**NiCOL**	Phase I	Nivolumab	Locally advanced cervical cancer	ORR: 93.8%; 2-year PFS: 75%; better immune responses correlated with improved outcomes.	Completed
**GOG-9929**	Phase I	Ipilimumab	Node-positive, HPV-related cervical cancer	Significant T-cell expansion and activation; poorer PFS linked to elevated tumor-promoting cytokines (e.g., TNFα, IL6).	Completed
**COLIBRI**	Phase II	Nivolumab + Ipilimumab	Locally advanced cervical SCC (FIGO IB3–IVA)	complete response rate: 82.5%; progression rate: 10%; minimal grade ≥3 AEs.	Completed
**ATOMICC**	Phase II	Dostarlimab	High-risk cervical cancer (advanced stages, para-aortic LN involvement)	evaluating maintenance therapy for relapse prevention after CRT.	Ongoing
**NRG-GY017/ATEZOLACC**	Phase I/Ib/II	Atezolizumab	High-risk cervical cancer	Promising immune activation and clinical benefits as a maintenance therapy after CRT.	Ongoing
**NACI trial**	Phase II	Camrelizumab + chemo	Locally advanced cervical cancer	ORR: 98%; CR: 19%; manageable AEs (e.g., lymphopenia 25%, neutropenia 12%); responders proceeded to surgery; others underwent CRT.	Completed
**JGOG trial**	Phase III	Z-100	Locally advanced cervical cancer	Trend toward improved OS (75.7% vs. 65.8%); not statistically significant (P = 0.07).	Completed
**NCT02247232**	Phase III	Z-100	Locally advanced cervical cancer	exploring overall survival benefits, with OS as the primary endpoint.	Ongoing

CALLA, Study of Durvalumab With Chemoradiotherapy for Women With Locally Advanced Cervical Cancer. KEYNOTE-A18, Study of Chemoradiotherapy With or Without Pembrolizumab for the Treatment of High-risk, Locally Advanced Cervical Cancer. NiCOL, Nivolumab in Association With Radiotherapy and Cisplatin in Locally Advanced Cervical Cancers Followed by Adjuvant Nivolumab for up to 6 Months. GOG-9929, Immune Activation in Patients with Locally Advanced Cervical Cancer Treated with Ipilimumab Following Definitive Chemoradiation. COLIBRI, COL Immunotherapy Before Radiochimio + Ipilimumab. ATOMICC, Trial of Anti-PD1, TSR-042, as Maintenance Therapy for Patients With High-risk Locally Advanced Cervical Cancer After Chemo-radiation. NRG-GY017/ATEZOLACC, Atezolizumab in Locally Advanced Cervical Cancer. NACI, Neoadjuvant Chemotherapy Plus Camrelizumab. JGOG, Japanese Gynecologic Oncology Group trial. NCT02247232, Randomized Study of Z-100 Plus Radiation Therapy to Treat Cervical Cancer.

Despite the promising theoretical foundation for durvalumab’s efficacy, the trial ultimately failed to demonstrate a significant improvement in PFS in the overall patient population. Although the combination of durvalumab and CRT was well tolerated, a deeper analysis revealed that PD-L1 expression might play a crucial role in determining which patients are more likely to benefit from this treatment. In contrast, the ENGOT-cx11/GOG-3047/KEYNOTE-A18 trial, which investigated pembrolizumab (200 mg for 5 cycles, followed by 400 mg for 15 cycles) in combination with CRT, yielded more promising results (109). This Phase 3 trial enrolled a cohort of 1,060 high-risk, locally advanced cervical cancer patients across 176 centers in 30 countries. The study demonstrated a 24-month PFS rate of 68% in the pembrolizumab arm, compared to 57% in the placebo group (HR = 0.70, p = 0.0020), although the OS difference at 24 months (87% vs. 81%) did not reach statistical significance. Importantly, adverse events of grade 3 or higher were more common in the pembrolizumab group (75% vs. 69%), suggesting that while the therapy has potential, close monitoring of adverse effects is warranted. Despite the modest impact on OS, these results highlight pembrolizumab’s potential to improve PFS in high-risk cervical cancer patients, positioning it as a key candidate for further clinical exploration. This combination has already been incorporated into the updated 2025 National Comprehensive Cancer Network (NCCN) guidelines, where pembrolizumab is recommended as part of the standard treatment regimen for FIGO 2014 and 2018 stage III–IVA cervical cancer. However, it should be noted that this approach has not yet been adopted by the European Society for Medical Oncology (ESMO) or the European Society of Gynecological Oncology, in collaboration with the European Society for Radiotherapy and Oncology and the European Society of Pathology (ESGO/ESTRO/ESP) (110, 111).

In addition to pembrolizumab, other immune checkpoint inhibitors are emerging as promising options in the treatment of cervical cancer. Nivolumab, an anti-PD-1 antibody, was evaluated in the NiCOL Phase 1 trial in combination with CRT (112). This trial included 16 patients with locally advanced cervical cancer, revealing an overall response rate (ORR) of 93.8%, alongside a two-year PFS rate of 75%. Immune response analysis from the study revealed that patients with better PFS exhibited stronger immune cell infiltrates and more robust immune cell interactions, whereas those with progressive disease had higher levels of PD-L1 expression and increased regulatory T cell populations. These findings suggest that nivolumab may be particularly beneficial for patients with an activated anti-tumor immune response, further supporting its potential in the treatment of cervical cancer.

Furthermore, the combination of ipilimumab, an anti-CTLA-4 antibody, with CRT has also shown promise in early-phase trials. The GOG-9929 Phase I trial (113), which tested this combination in 21 patients with node-positive, HPV-related cervical cancer, demonstrated significant T-cell population expansion and upregulation of activation markers, such as ICOS and PD-1, particularly enhancing HPV-specific T-cell responses in HPV18+ tumors. However, elevated levels of tumor-promoting cytokines, including TNFα and IL6, were associated with poorer PFS, emphasizing the need for precise modulation of immune responses to optimize clinical outcomes. In a related Phase II neoadjuvant study, the COLIBRI trial combined nivolumab and ipilimumab in locally advanced cervical squamous cell carcinoma (FIGO stages IB3–IVA), resulting in impressive outcomes such as significant CD8+ T-cell infiltration, high complete response rates (82.5%), and low progression rates (10%), particularly in stage III-C disease (114). These promising results suggest that dual immune checkpoint blockade may enhance anti-tumor immunity in high-risk cervical cancer patients.

In parallel, maintenance immunotherapy strategies are under active investigation as a means to prolong PFS and reduce the risk of recurrence. The ongoing ATOMICC trial is assessing dostarlimab, an anti-PD-1 checkpoint inhibitor, as a maintenance therapy for patients who achieved partial or complete responses following CRT (115). This Phase II trial focuses on high-risk cervical cancer patients, particularly those with advanced FIGO stages or para-aortic lymph node involvement, to determine whether dostarlimab can effectively reduce relapse rates. Likewise, atezolizumab, a PD-L1 inhibitor, is being explored in combination with CRT in several trials, including the NRG-GY017 Phase I/Ib and ATEZOLACC Phase II studies, which have demonstrated promising immune activation and potential clinical benefits. These findings position atezolizumab as a potentially valuable maintenance option in cervical cancer.

Neoadjuvant and adjuvant approaches, such as the combination of Camrelizumab, an anti-PD-1 monoclonal antibody, with chemotherapy, are also being explored. A Phase II trial conducted across multiple centers in China reported an ORR of 98%, with 19% of patients achieving a complete response (116). Patients who responded to this chemo-immunotherapy combination proceeded to surgery, while non-responders underwent CRT. While the treatment was generally well tolerated, the most common grade 3–4 adverse events were lymphopenia (25%) and neutropenia (12%), underscoring the need for careful management of immune-related toxicities. Additionally, Z-100, an extract, derived from *Mycobacterium tuberculosis*, which restores the Th1 immune response (117), has been evaluated in a Phase III trial by the Japanese Gynecologic Oncology Group. Although the trial did not reach statistical significance, there was a trend toward improved overall survival in the Z-100 group (75.7%) compared to the placebo group (65.8%), indicating its potential to enhance the efficacy of radiotherapy (118). Ongoing trials, such as NCT02247232, are expected to provide further insights, with overall survival as the primary endpoint.

The ICIs’ mechanism of action is through the reactivation of anti-tumor T-cells, resulting in a new tumor cytoxicity profile with immunotherapy-related adverse effects significantly different from those of conventional chemotherapy. In a recent retrospective study, Shehaj et al., evaluated the occurrence and type of immune-associated side effects in a cohort of 61 patients with gynecological malignancies who received ICIs; anti–PD-1 antibodies (pembrolizumab, dostarlimab, durvalumab) or the anti-PD-L1 antibody atezolizumab (119). The key finding is that ICIs therapy was well tolerated, with anti-PD-(L)1 antibodies tending to be better tolerated (120). The duration of ICI therapy was reported as the only significant factor influencing the incidence of adverse events (119).

Feng et al., showed that adverse events did not differ between the first-line ICIs plus platinum and paclitaxel group versus first-line platinum and paclitaxel group in advanced and recurrent cervical cancer patients (121). The common reported adverse effects of Pembrolizumab are tolerable liver dysfunction, hypothyroidism, neutropenia, anemia, decreased appetite, fatigue, and fever. Most of them improved after therapy discontinuation (122). A few cases of cutaneous adverse events were reported associated with pembrolizumab combination chemotherapy in patients with metastatic or recurrent cervical cancer (123). Adverse events related to nivolumab and atezolizumab, PD-1/PD-L1 inhibitors still at I/II phase clinical trial, are unavailable (122).

The high cost of immunotherapies constitute a financial burden, limiting patients’ access to these treatments (124). Immunotherapy cost-effectiveness vary significantly among countries and depends on the treatment indication and the use of biomarkers, such as PDL1 (125). Till now, no clear evidence regarding the cost-effectiveness of anti PD-L1 and PD-1 for treating LACC have been presented, and available data are not in favor of the cost-effectiveness of anti PD-L1 in LACC (126). Only one cost-effectiveness analysis for the treatment of patients with cervical cancers was conducted through a partitioned survival model indicating that pembrolizumab was not cost-effective versus the placebo (127). The cost is a major barrier to immunotherapy accessibility in low- and middle-income countries. Studies investigating the most cost-effective dosing strategies for ICIs are urgently needed (124). Furthermore, healthcare systems must focus on improving prevention programs, which offer the most cost-effective strategy for the control of cervical cancer.

Finally, patient selection for immunotherapy approaches remains challenging. Further research and new guidelines are necessary to address the best personalized therapeutic options based on histological subtype, PD-L1 status, and patient’s relapse history as they are no longer ICI-naïve. Thus, research on predictive biomarkers using innovative approaches, has to be strengthened. The focus should also be on the best sequencing immunotherapy strategies: neo-adjuvant, concomitant, maintenance, or a combination; monotherapy or dual immune checkpoint inhibitors; and the combination of chemotherapy with immunotherapy. Furthermore, with the increased use of immunotherapies, how to overcome resistance is a major concern. The presence of primary mechanisms of resistance deprive patients of immunotherapy benefits. Deciphering the intricate TME and its dynamics will enable a better patient’s stratification and thus more precise and effective immunotherapy.

## 7 Conclusion

Immune checkpoint inhibitors represent a paradigm shift in the treatment of locally advanced cervical cancer. While some studies highlight the complexities of patient selection, others reinforce the potential of checkpoint blockade in enhancing PFS. As additional data emerge, optimizing patient stratification through biomarkers, refining combination strategies, and addressing immune-related toxicities will be critical in integrating immunotherapy into standard cervical cancer treatment protocols. Furthermore, the development of novel agents, exploration of synergistic therapeutic combinations, and ongoing refinement of treatment sequencing will play a pivotal role in maximizing clinical benefits. Future research should focus on identifying predictive biomarkers, improving patient selection criteria, and mitigating adverse effects to ensure that immunotherapy is both effective and tolerable for a broader patient population.
